# Decomposition of socioeconomic inequalities in the uptake of intermittent preventive treatment of malaria in pregnancy in Nigeria: evidence from Demographic Health Survey

**DOI:** 10.1186/s12936-021-03834-8

**Published:** 2021-07-03

**Authors:** Chijioke Ifeanyi Okoli, Mohammad Hajizadeh, Mohammad Mafizur Rahman, Rasheda Khanam

**Affiliations:** 1 School of Business, and Centre for Health Research, University of Southern Queensland, Toowoomba, QLD 4350 Australia; 2 Department of Health Administration and Management, Faculty of Health Sciences and Technology, College of Medicine, University of Nigeria, Enugu Campus, Enugu, Enugu State Nigeria; 3 School of Health Administration, Dalhousie University, Halifax, Canada

**Keywords:** Socioeconomic, Inequalities, Concentration index, Decomposition analysis, Malaria, Intermittent preventive treatment in pregnancy, Nigeria

## Abstract

**Background:**

Although malaria in pregnancy is preventable with the use of intermittent preventive treatment with sulfadoxine–pyrimethamine (IPTp-SP), it still causes maternal morbidity and mortality, in sub-Saharan Africa and Nigeria in particular. Socioeconomic inequality leads to limited uptake of IPTp-SP by pregnant women and is, therefore, a public health challenge in Nigeria. This study aimed to measure and identify factors explaining socioeconomic inequality in the uptake of IPTp-SP in Nigeria.

**Methods:**

The study re-analysed dataset of 12,294 women aged 15–49 years from 2018 Nigeria Demographic Health Survey (DHS). The normalized concentration index (C_n_) and concentration curve were used to quantify and graphically present socioeconomic inequalities in the uptake of IPTp-SP among pregnant women in Nigeria. The C_n_ was decomposed to identify key factors contributing to the observed socioeconomic inequality in the uptake of adequate (≥ 3) IPTp-SP.

**Results:**

The study showed a higher concentration of the adequate uptake of IPTp-SP among socioeconomically advantaged women (C_n_ = 0.062; 95% confidence interval [CI] 0.048 to 0.076) in Nigeria. There is a pro-rich inequality in the uptake of IPTp-SP in urban areas (C_n_ = 0.283; 95%CI 0.279 to 0.288). In contrast, a pro-poor inequality in the uptake of IPTp-SP was observed in rural areas (C_n_ = − 0.238; 95%CI − 0.242 to − 0.235). The result of the decomposition analysis indicated that geographic zone of residence and antenatal visits were the two main drivers for the concentration of the uptake of IPTp-SP among wealthier pregnant women in Nigeria.

**Conclusion:**

The pro-rich inequalities in the uptake of IPTp-SP among pregnant women in Nigeria, particularly in urban areas, warrant further attention. Strategies to improve the uptake of IPTp-SP among women residing in socioeconomically disadvantaged geographic zones (North-East and North-West) and improving antenatal visits among the poor women may reduce pro-rich inequality in the uptake of IPTp-SP among pregnant women in Nigeria.

**Supplementary Information:**

The online version contains supplementary material available at 10.1186/s12936-021-03834-8.

## Background

Malaria in pregnancy (MiP) is a global public health concern with severe consequences for the mother, the foetus, and the newborn child [1–3]. In 2018, an estimated 219 million cases and 435,000 deaths from malaria were recorded globally and about 80% of these deaths were reported by the World Health Organization (WHO) African Region and India [1, 4]. MiP is an obstetric, medical, social, and economic, emergency that is preventable and/or treatable but still causes maternal morbidity and mortality in sub-Saharan Africa [5–7]. Despite this evidence and the ongoing efforts to eliminate malaria, the use of intermittent preventive treatment in pregnancy (IPTp) is still insufficient [3, 6, 8].

IPTp with sulfadoxine–pyrimethamine (IPTp-SP) has proven to be efficacious in reducing the amount of MiP [9]. However, although, the WHO recommends that all pregnant women living in malaria-endemic regions take at least 3 doses of IPTp-SP during their pregnancy, studies have shown poor uptake among pregnant women in many areas [10]. For many countries in sub-Saharan Africa, access to and use of these interventions by pregnant women is low and achievement of high coverage among pregnant women remains elusive [11].

In 2001, Nigeria instituted IPTp-SP for pregnant women in the second and third trimesters of pregnancy. However, twenty years later both first and second dose coverage remains low being 8.0% and 4.6%, respectively [12, 13]. The use of IPTp in Nigeria involves the administration of at least two curative doses of SP during pregnancy, regardless of whether the woman is infected [12, 13]. Previous studies in Nigeria show variations in uptake of IPTp-SP across the states in Nigeria [1, 6]. Low uptake of IPTp-SP can lead to high malaria cases among pregnant women, which could culminate in low birth weights, a higher number of stillbirths, spontaneous abortion, asymptomatic infection, with potential results being maternal anaemia and placental parasitaemia, premature delivery, and maternal and infant deaths [5].

Several studies indicate that socioeconomic inequalities prevail in the IPTp-SP uptake in Nigeria [1, 6, 9, 12, 14, 15]. For example, Akpa and colleagues in their work in select communities of Ebonyi State in Nigeria found that women whose husbands had secondary and tertiary education were more likely to have IPTp-SP uptake than those whose husbands had below secondary education [1]. As the study by Alawode and colleagues asserts, there are significant differences in access to some of the malaria interventions that favour the better off in society as a whole and some geopolitical regions in particular [15].

This study aim to measure socioeconomic inequality in the uptake of IPTp-SP in Nigeria. As a way of measuring this, a decomposition analysis is used to identify factors that contribute to the socioeconomic inequality in the uptake of IPTp-SP in Nigeria. The results of this study provide evidence for intervention to reduce socioeconomic inequality in the uptake of IPTp-SP in Nigeria. The study is in tandem with the Sustainable Development Goal 3 targets to reduce the global maternal mortality ratio to less than 70 per 100,000 live births and reduce neonatal mortality to as low as 12 per 1000 live births by 2030 [16].

## Methods

### Study setting

The study focuses on Nigeria. Nigeria, with an estimated population of 198 million in 2018, is the most populous country in Africa [17]. The population is predominantly young, with about 45% aged under 15 years and 20% under 5 years, while women of childbearing age (15–49 years) account for about 22% of the total population [18]. The country is divided into six geopolitical zones: North-Central, North-East, North-West, South-East, South-South, and South-West with each geopolitical zone comprising about six states [19]. Of the six zones, the northern geopolitical zones especially the North West and North East have the highest poverty rates in the country [20]. The health care system in Nigeria is largely public sector driven, with substantial private sector involvement in service provision. Most secondary- and tertiary-level health facilities are in urban areas, whereas rural areas are predominantly served by primary health care (PHC) facilities. There is a shortage of PHC facilities in some states [18] and less than 20% of health facilities in the country offer emergency obstetric care, despite that Nigeria accounts for one-quarter of all malaria cases in Africa [17]. Malaria accounts for 60% of outpatient visits and 30% of hospitalizations among children under five years of age in Nigeria. The malaria prevalence among children 6–59 months in the six geopolitical zones was as follows, South West (50.3%), North Central (49.4%), North West (48.2%), South-South (32.2%), North East (30.9%) and South East (27.6%) [21].

### Data

Data for the analyses were obtained from the latest Nigeria Demographic Health Survey (DHS) 2018, conducted between August 14, 2018 to December 29, 2018 [22]. The choice of the dataset is to ensure that the research finding represents current reality, which is nationally representative, and generalizable. The dataset contains survey information elicited from the women of reproductive age of 15–49 years in the six geopolitical zones in the country. The study used an Individual (women) Recode file that collected information on women’s and husband’s background characteristics, reproductive history, antenatal care, malaria prevention and treatment, household asset ownership, and type of toilet facilities among other information. The DHS survey uses a multistage sampling procedure, standardized tools and well-trained interviewers to collect comparable and reliable data on maternal and child health. The 2018 Nigeria DHS had a response rate of 99%. Further information about the survey has been provided elsewhere [17, 22]. The analysis of this study was restricted to all pregnant women age 15–49 in the women sample (n = 13,705). After dropping 227 and 1184 observations with missing information in the outcome and independent variables respectively, the final sample size for the study comprised 12,294 pregnant women.

### Variables

#### Outcome variable

The dependent variable is the uptake of adequate (≥ 3) IPTp-SP, categorized following the WHO recommendation of IPTp-SP doses, women took during pregnancy in the year preceding the surveys. The variable was categorized as less than three doses of IPTp-SP, as inadequate uptake (i.e. < 3 doses = 0), and at least three doses or more ≥ 3 doses = 1, as adequate uptake [1, 6, 23].

#### Socioeconomic status

The wealth index variable was used as a proxy for socioeconomic status. It was constructed using household ownership of selected assets (e.g. televisions and bicycles), materials used in housing construction, type of water access, and sanitation facilities data via a principal component analysis (PCA) [24].

#### Independent variables

Based on the current literature [6, 9, 23, 25, 26], the following variables were used as determinants of the uptake of IPTp-SP among pregnant women: age groups, the level of education, marital status, religion, occupation, place of residence (rural and urban), geopolitical zone, wealth index quintiles, husband/spouse level of education, distance to a health facility, and a requirement to obtain permission for self-medical help (defined as either a big problem or not a big problem) to access IPTp-SP [6, 23]. Additional file 1: Table S1 reports description of variables used in the analysis.

### Statistical analysis

#### Measuring socioeconomic inequalities in the uptake of IPTp-SP

This study used the concentration index (C) approach, an appropriate and most widely used measure of socioeconomic-related inequality [27–29], to quantify socioeconomic inequalities in IPTp-SP. The C is based on the concentration curve which graphs the cumulative share of the population on the x-axis and the cumulative share of the health outcome on the y-axis. The C index is defined as twice the area between the concentration curve and the line of perfect equality (45-degree diagonal) [29, 30]. The C can be computed using the convenient regression method as follows:

1 $$2\sigma _{r}^{2} \left( {\frac{{h_{i} }}{\mu }} \right) = \alpha + \beta r_{i} + \varepsilon _{i} ,$$

where ${\sigma }_{r}^{2}$ is the variance of the fractional rank, $h$ is the healthcare variable of interest (i.e. IPTp-SP uptake) of $i$ th woman, $\mu$ is the mean of the variable of interest, h, for the whole population, and ${r}_{i}=\frac{1}{N}$ is the fractional rank of the $i$ th woman in the distribution of socio-economic position, with $i=1$ for the poorest and $i=N$ for the richest. The C is calculated as the ordinary least squares (OLS) estimate of $\beta$ [29, 31].

The C ranges from − 1 to + 1, for continuous health outcomes. Since the outcome variable (IPTp-SP) of interest is binary, the minimum and maximum of the C are not between − 1 and + 1 but depend on $\mu$ [32]. Hence, the index can be normalized by multiplying the estimated C by $\frac{1}{1-\mu }$. The normalized concentration (C_n_) index is used to quantify socioeconomic-related inequalities in uptake of adequate (≥ 3) IPTp-SP. If the value of the C_n_ is zero, it suggests that there is no socioeconomic-related inequality in health outcomes. A negative (positive) value of the C_n_ when the curve lies above (below) the line of equality indicates a disproportionate concentration of the health variable (i.e. IPTp-SP) among the poor (rich) [28, 29]. A higher value of the C_n_ corresponds to high socioeconomic inequalities [27].

#### Decomposition analysis

The C_n_ is decomposed to quantify factors (demographic, geographic, and socioeconomic) that contribute to the observed socioeconomic inequalities in the uptake of adequate IPTp-SP following the Wagstaff, Van Doorslaer [33] approach. If there is a linear regression model to link the outcome variable (i.e. uptake of adequate IPTp-SP) $h$, to a set of $k$ explanatory factors, ${x}_{\kappa }$ such as:

2 $$h = \alpha + \mathop \sum \limits_{\kappa } \beta _{\kappa } x_{\kappa } + ~\varepsilon .$$

where $\alpha$ and $\beta$ are parameters that measure the relationship between each explanatory factor $x$ and the uptake of adequate IPTp-SP and $\varepsilon$ error term.

Wagstaff, Van Doorslaer [33] showed that the C of $h$, can be decomposed into the contribution of determinants that explain the uptake of IPTp-SP during pregnancy as follows:

3 $$C = \sum\limits_{k} {\left( {\frac{{\beta _{k} \overline{{\chi _{k} }} }}{\mu }} \right)} C_{K} + ~\frac{{GC_{\varepsilon } }}{\mu }$$

where $\stackrel{-}{x}$_k_ is the mean of ${x}_{k}$, and ${C}_{k}$ denotes the concentration index for ${x}_{k}$, a contributing factor. The ${GC}_{\varepsilon }$ denotes the generalized concentration index of the error term, ${\varepsilon }_{i}$.

Equation 3 shows that the overall inequality in the uptake of IPTp-SP has two components. The first term $(\frac{{\beta }_{k}\stackrel{-}{{x}_{k}}}{\mu }){C}_{K}$ denotes the contribution of factor k to socioeconomic inequality in the uptake of adequate IPTp-SP. It constitutes the deterministic or explained component of the IPTp-SP uptake of the concentration index. The second term $\frac{{GC}_{\varepsilon }}{\mu }$ represents the unexplained component or the residual of the IPTp-Sp uptake [31, 34]. Based on Eq. 3, the product of the elasticity of each factor and its ${C}_{k}$ gives the contribution of that factor to the inequality. The negative (positive) contribution of a predictor to the ${C}_{n}$ suggests that the socioeconomic distribution of the predictor and the association between the predictor and the adequate uptake of IPTp-SP leads to an increase in the concentration of uptake of IPTp-SP among the poor (rich). A zero value of either elasticity or the ${C}_{k}$ leads to the zero contribution of the factor to C [29, 35].

Applying the Wagstaff [32] normalization approach to the decomposition of the C can yield:

4 $$C_{n} = \frac{C}{{1 - \mu }} = \frac{{\mathop \sum \nolimits_{k} \left( {\frac{{\beta _{k} \overline{{x_{k} }} }}{\mu }} \right)C_{K} }}{{1 - \mu }} + \frac{{\frac{{GC_{\varepsilon } }}{\mu }}}{{1 - \mu }}$$

The dataset was weighted using the primary sampling weight provided in the DHS to obtain estimates that are representative of all pregnant women in Nigeria. A survey logistic estimation on samples was conducted to check for collinearity before the decomposition analysis. Chi-square was used to test associations between socio-demographic characteristics and IPTp-SP uptake. The predictors of IPTp-SP uptake were considered statistically significant at p < 0.05. All data analyses were conducted using Stata/SE-13 software [36].

## Results

### Descriptive statistics

Table 1 reports descriptive statistics of variables used in the study. The average age of women in the sample was 29.2 years old. Just over a quarter of pregnant women (27.3%) had adequate (i.e. ≥ 3) uptake of IPTp-SP during pregnancy. Approximately 67% of women in the sample were married. When education was measured, 35% of both women and spouses had no formal education, while 40% and 34% of the women and spouse had a secondary education level, respectively. Moreover, about 65% of the women were employed and 59.4% of them resided in rural areas. Interestingly, over half (57%) of the women received at least four ANC visits.

**Table 1:** Descriptive statistics of variables used in the study

	Mean or percentage
Variable	
Adequate uptake (≥ 3) of IPTp-SP during pregnancy	27.3%
Demographic variables	
Woman’s age	29.2
Marital status	
Married	66.5%
Others	33.5%
Socioeconomic variables	
Woman education level	
No formal education	34.6%
Primary education	15.3%
Secondary education	40.0%
Higher education	10.4%
Spouse education level	
No formal education	34.6%
Primary education	15.6%
Secondary education	34.1%
Higher education	15.7%
Wealth index	
Poorest (1)	18.6%
Poorer (2)	20.0%
Middle (3)	21.1%
Richer (4)	21.1%
Richest (5)	19.2%
Employment status	
Woman is employed	64.6%
Religion	
Christian	49.1%
Muslim	50.0%
Others	0.9%
Ecological variable	
Place of residence	
Urban residence	40.6%
Geopolitical zone	
North-Central	18.7%
North-East	18.4%
North-West	24.2%
South-East	13.3%
South-South	12.1%
South-West	13.3%
Distance to a health facility	
Distance to a clinic is a big problem	27.5%
Getting medical help for self	
Permission for self-medical help (big problem)	11.7%
Antenatal care variable	
Number of ANC visits	
≥ 4 times	57.2%
Sample size	12,294

*IPTp-SP* intermittent preventive treatment in pregnancy with sulfadoxine–pyrimethamine, *ANC* antenatal care

#### Socioeconomic inequality in adequate (≥ 3) uptake of IPTp-SP during pregnancy

**Fig. 1:**
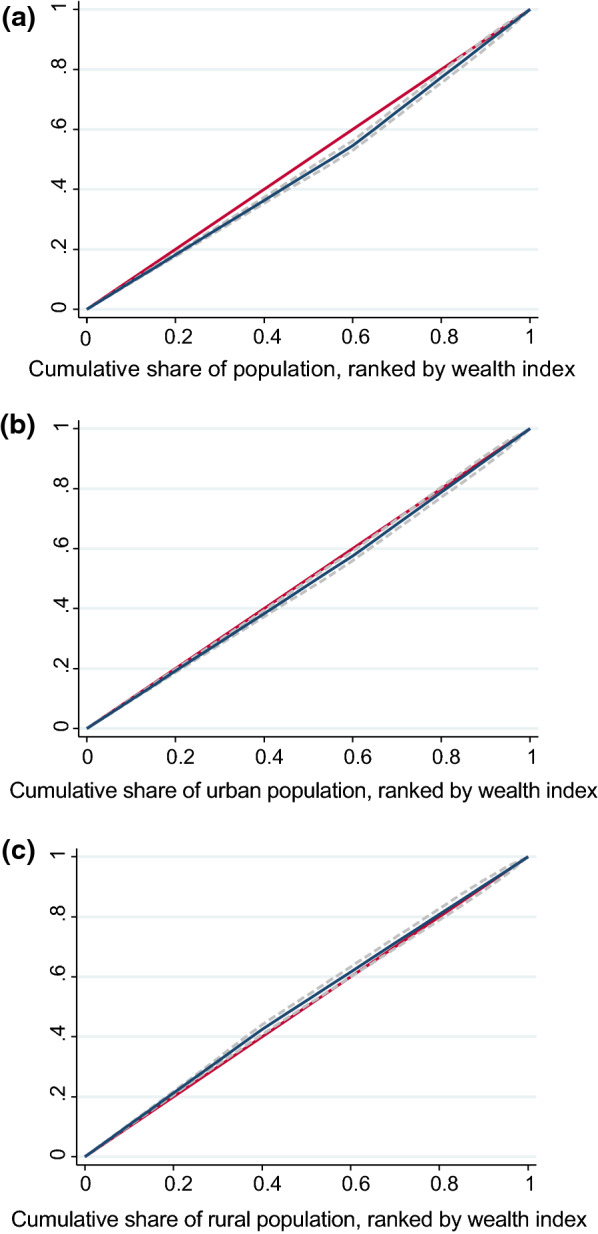
The concentration curve for adequate (≥ 3) uptake of Intermittent Preventive Treatment in Pregnancy with Sulfadoxine-pyrimethamine (IPTp-SP) in total, urban and rural areas of Nigeria. **a** Concentration curve of IPTp-SP uptake in Nigeria as a whole. **b** Concentration curve of IPTp-SP uptake in urban areas of Nigeria. **c** Concentration curve of IPTp-SP uptake in rural areas of Nigeria

Figure 1 reports the concentration curve for adequate (≥ 3) uptake of IPTp-SP by pregnant women in Nigeria, in urban and rural areas. The concentration curve for adequate (≥ 3) uptake of IPTp-SP for Nigeria as a whole and urban areas lies below the 45-degree diagonal line indicating that adequate uptake of IPTp-SP is concentrated among the wealthier women. However, the concentration curve of rural areas lies above the line of equality, suggesting that adequate uptake of IPTp-SP is concentrated among the poorer women.

Table 2 reports the C_n_ values for an adequate (≥ 3) uptake of IPTp-SP. Similar to the concentration curves, the C_n_ results suggested that adequate uptake of IPTp-SP in pregnancy is concentrated among socioeconomically advantaged women (C_n_ = 0.062; 95%CI 0.048 to 0.076) in the country, as well as in urban areas (C_n_ = 0.283; 95%CI 0.279 to 0.288). In contrast, pro-poor (favours the poor) inequality in uptake of IPTp-SP was found in the rural areas (C_n_ = − 0.238; 95%CI = − 0.242 to -0.235).

**Table 2:** Socioeconomic inequalities for adequate (≥ 3) uptake of IPTp-SP in pregnancy in Nigeria

The C_n_ Index		
Total	Urban	Rural
0.062 (0.048 to 0.076)	0.283 (0.279 to 0.288)	− 0.238 (− 0.242 to − 0.235)

95% confidence intervals in parentheses

#### Decomposition of the socio-economic inequality in adequate (≥ 3) uptake of IPTp-SP

Table 3 reports the decomposition results of the socioeconomic inequalities of adequate (≥ 3) uptake of IPTp-SP among pregnant women in 2018 in Nigeria. The table contains the estimated marginal effects of the predictor variables obtained from the logit model, the elasticities, the concentration index of the predictor variables (${C}_{k}$), and the contribution of each predictor variable to the C_n_. The elasticity column denotes the change in the outcome variable (i.e. adequate uptake of IPTp-SP) associated with a one-unit change in the independent variables. It represents the responsiveness of the health outcome i.e. adequate uptake of IPTp-SP to a change in the predictor variable. A negative (positive) sign in elasticity shows a decreasing (increasing) change of adequate uptake of ITPp-SP in association with a change in the predictor.

**Table 3:** Decomposition of the socioeconomic inequality in adequate (≥ 3) uptake of IPTp-SP among pregnant women in Nigeria, 2018

Variables	Marginal effect	Elasticities	Ck	Contribution to C_n_		Percentage contribution (%)
				Absolute	Summed	
Age group						
15–24 (ref)						
25–34	0.007***	0.009	0.017	0.000		
35–49	0.010***	0.012	0.007	0.000		
Marital status						
Married	0.012***	0.031	− 0.059	− 0.001	− 0.001	− 1.90
Others (ref)						
Level of education (women)						
No formal education (ref)						
Primary	− 0.017***	− 0.009	− 0.096	0.001		
Secondary	− 0.015***	− 0.023	0.197	− 0.003		
Tertiary	− 0.021***	− 0.009	0.459	− 0.003		
Level of education (husband)						
No formal education (ref)						
Primary	0.003***	0.002	− 0.097	0.000		
Secondary	0.052**	0.067	0.173	0.008		
Tertiary	0.054**	0.033	0.395	0.010	0.018	20.01
Wealth index of households						
Poorest (ref)						
Poorer	− 0.075*	− 0.056	− 0.437	0.018		
Middle	− 0.091*	− 0.068	− 0.437	0.022		
Richer	− 0.078*	− 0.064	0.563	− 0.026		
Richest	− 0.083*	− 0.070	0.563	− 0.029	− 0.016	− 25.90
Employment status						
Unemployed (ref)						
Employed	− 0.015***	− 0.038	0.018	− 0.001	− 0.001	− 0.84
Religion						
Christian (ref)						
Muslim	0.035***	0.072	− 0.129	− 0.007		
Others	− 0.139	− 0.003	− 0.147	0.000	− 0.006	− 10.57
Place of residence						
Urban	− 0.024***	− 0.042	0.282	− 0.009	− 0.009	− 14.28
Rural (ref)						
Geopolitical zone						
North-Central (ref)						
North-East	− 0.073*	− 0.045	− 0.235	0.008		
North-West	− 0.091*	− 0.102	− 0.206	0.016		
South-East	0.249	0.113	0.180	0.015		
South-South	0.070*	0.031	0.228	0.005		
South-West	− 0.014***	− 0.009	0.351	− 0.002	0.041	67.38
Distance to health facility (big problem)	− 0.029**	− 0.029	− 0.175	0.004	0.004	6.17
Permission for self-medical help (big problem)	0.005***	0.002	− 0.106	0.000	0.000	− 0.31
Number of ANC visits (≥ 4 times)	0.068*	0.151	0.148	0.016	0.016	26.92
Sum					0.041	66.67
Residual					0.020	33.33
Total Cn					0.061	100.00

The percentage of contributions was calculated by dividing the specific “summed” contribution by the absolute values of C_n_ and multiplying by 100. The sum of all the percentage contributions should add up to 100 percent. The value 0.00 is not zero but due to rounding; Marginal effects were calculated at the means of the predictor variables

*IPTp-SP* intermittent preventive treatment in pregnancy with sulfadoxine–pyrimethamine, *ANC* antenatal care

*** p < 0.01, ** p < 0.05, * p < 0.1

The negative (positive) sign of the ${C}_{k}$ for a certain variable suggests that the predictor concentrated among the poor (rich) individuals. For instance, as reported in the table, the marital status of the married, primary educated women of the Muslim religion, was concentrated among the poor, whereas secondary and tertiary education, urban residence, and the number of antenatal care visits (≥ 4) were more concentrated among the rich.

The estimated contribution of predictors to the ${C}_{n}$ suggested that demographic factors, level of education for women, wealth index, employment status, religion, and urban place of residence contributed negatively to socioeconomic inequality in the uptake of IPTp-SP in 2018 in Nigeria. In contrast, the level of education for husbands, the geopolitical zones, distance to a health facility, and the number of antenatal visits of four and above positively contributed to socioeconomic inequality for adequate uptake of IPTp-SP in 2018 in the country.

**Fig. 2:**
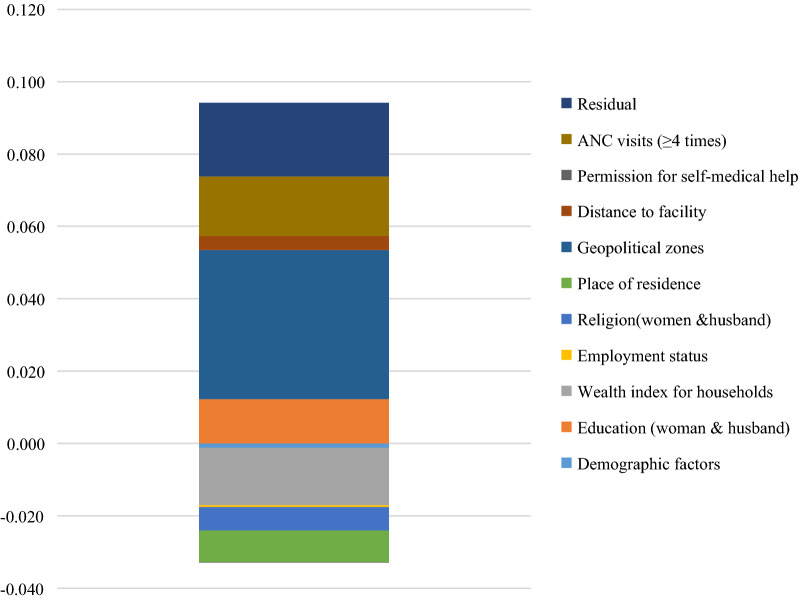
Absolute contribution of each factor to socioeconomic inequality in the uptake of IPTp-SP in Nigeria, 2018. The y-axis of the chart shows the absolute negative/positive contribution of each predictor to the C_n_; *IPTp-SP* Intermittent preventive treatment in pregnancy with sulfadoxine–pyrimethamine, *ANC* antenatal care

Figure 2 illustrates the absolute contribution of a predictor to the socioeconomic inequalities for adequate uptake of IPTp-SP in 2018 in Nigeria. As reported in Table 3 and shown in Fig. 2, geopolitical zone (67.4%), education (20.01%), antenatal care visits of four and above (26.9%), and distance to health facility (6.2%) were the most important predictors contributing to and/or explained the observed pro-rich (favours the rich) inequalities in uptake of IPTp-SP in the country. However, the wealth index (− 25.9%), place of residence (− 14.3%), and religion (− 10.6%), amongst others contributed negatively to the socioeconomic inequality for adequate uptake of IPTp-SP.

The results suggested the independent variables included in the model explained a sum of 66.7% of the observed socioeconomic inequality for adequate uptake of IPTp-SP among pregnant women in 2018. A substantial contribution of the unexplained or residual component 33.3% implies that there are predictors other than the variables in the model that affect adequate uptake of IPTp-SP in pregnancy in Nigeria, which could be not observed, or controlled in the this study.

## Discussion

This study sought to measure and decompose socioeconomic inequality in the uptake of IPTp-SP in Nigeria. Monitoring health inequalities helps countries to track their progress towards the Sustainable Development Goals and ensures that vulnerable populations are not overlooked [37]. Decomposing socioeconomic inequalities in health helps to uncover specific factors that are modifiable by policymakers [38].

The descriptive results demonstrated a low uptake of IPTp-SP (27.3%) in Nigeria, notwithstanding that over half (57%) of the women attended at least four ANC visits and above. This indicates that high ANC attendance alone is not sufficient to ensure high uptake of IPTp-SP due to operational challenges of service delivery (staff shortages, drug stock-out, and poor health worker attitude/remuneration) [25], and lack of knowledge of prophylaxis for malaria prevention [25]. Thus, there is a need to launch a targeted promotional campaign to reach the vulnerable population of pregnant women [11].

Moreover, our findings indicated pro-rich inequalities in the uptake of IPTp-SP among pregnant women in Nigeria, especially in the urban area. There are substantial socioeconomic inequalities across urban settings in Nigeria. The existence of such inequalities indicates that many urban dwellers (especially the urban-poor and urban-middle) do not have access to key resources and services, unlike the urban-rich [39]. The inequality reduces opportunities among the urban-poor, underserved, and vulnerable groups due to fierce competition for available resources, which only the ‘fittest’ so to say wins [39]. However, this seems not to be the case in rural areas as the poor use more IPTp-SP than the rich, contrary to expectation. As maternal health services in Nigeria were considered of poor quality [40], the better off compared to the less well-off women may use fewer IPTp-SP in rural areas because health care demand of the poor is less sensitive to quality than that of the better off [41]. Hence, there is a need to improve provider practices for IPTp-SP delivery in Nigeria [13], especially in rural settings. The higher uptake of IPTp-SP among the poor women in rural areas may also be due to differences in the assessment of malaria exposure risk between the rich and the poor women in Nigeria. In other words, poor pregnant women in rural areas may consider themselves at higher risk of contracting malaria; thus, they use more IPTp-SP than the rich.

The geopolitical zone of residence, an education level (spouse), antenatal care visits of four and above, and distance to health facilities were found to be the main drivers' of pro-rich inequalities in the uptake of IPTp-SP in Nigeria. Education level (spouse) and antenatal care visit contributed to the concentration of the uptake of IPTp-SP in Nigeria among the rich because these factors, on the one hand, are positively associated with the uptake of IPTp-SP among pregnant women, and on the other hand, they are more concentrated among wealthier women. The positive impacts of the level of education (spouse) and ANC visits on adequate uptake of IPTp-SP were also found in the previous studies [23, 27]. Geopolitical zone of residence contributed to the pro-rich inequalities in the uptake of IPTp-SP because pregnant women in the two socioeconomically disadvantaged geographic zones of North-East and North-West consumed fewer IPTp-SP compared to other geographic zones. It is interesting to note that the household wealth index contributed negatively to socioeconomic inequality in the uptake of IPTp-SP in Nigeria. This could be explained by the fact that IPTp-SP is administered free at health facilities in Nigeria and, therefore, poor women do not have a financial barrier of access to IPTp-SP.

The decomposition analysis also showed a significant contribution of the residual (i.e., the unexplained portion of the model) to the observed pro-rich inequality in the uptake of IPTp-SP in Nigeria. The distribution of some of the omitted health systems factors (supply-side) from the model might have contributed to the pro-rich inequalities in the uptake of IPTp-SP among pregnant women. In other words, poor service delivery for low socioeconomic status (SES) pregnant women in the country may have reduced the uptake of IPTp-SP in these groups, as the quality of service is one of the greatest barriers to utilizing maternal healthcare [18, 27].

This study is subject to some limitations. Firstly owing to the unavailability of some data in the DHS, we could not include supply-side factors such as patient satisfaction/quality of service delivery variables in the decomposition analysis. In the same vein, other confounding factors such as pregnancy mortality, preterm delivery, parity and HIV infection that could influence IPTp-Sp uptake were unaccounted for due to unavailability of data. Thus, further research should be undertaken to examine, especially the influence of supply-side factors on socioeconomic inequality and adequate uptake of IPTp-SP using a mixed-method. Secondly, the DHS data on IPTp-SP uptake was based on self-report elicited from pregnant women and as such may introduce a systematic error like recall bias.

## Conclusion

This study demonstrated pro-rich inequalities in the uptake of IPTp-SP among pregnant women in Nigeria. The concentration of the uptake of IPTp-SP among wealthier pregnant women in urban areas is particularly concerning. Thus, there is a need to improve the uptake of IPTp-SP among women residing in socioeconomically disadvantaged geographic zones (especially in the North-East and North-West) and increased access to ANC among poor women to mitigate pro-rich inequality in the uptake of IPTp-SP in Nigeria.

## Supplementary Information

**Additional file 1: Table S1.** Description of variables used in the study.

## Data Availability

Data for this study is publicly accessible at the DHS website: https://www.dhsprogram.com/data/available-datasets.cfm

## Abbreviations

ANC: Antenatal care
CI: Concentration index
DHS: Demographic Health Survey
IPTp-SP: Intermittent preventive treatment with sulfadoxine–pyrimethamine (IPTp-SP)
MiP: Malaria in pregnancy
PHC: Primary health care
PCA: Principal component analysis
SSA: Sub-Saharan Africa
WHO: World Health Organization

## References

[1] Akpa CO, Akinyemi JO, Umeokonkwo CD, Bamgboye EA, Dahiru T, Adebowale AS (2019). Uptake of intermittent preventive treatment for malaria in pregnancy among women in selected communities of Ebonyi State, Nigeria. BMC Pregnancy Childbirth.

[2] Florey L. Preventing malaria during pregnancy in sub-Saharan Africa: determinants of effective IPTp delivery. In: DHS Analytical studies No 39. Maryland: ICF International; 2013.

[3] Yaya S, Uthman OA, Amouzou A, Bishwajit G (2018). Use of intermittent preventive treatment among pregnant women in sub-Saharan Africa: evidence from malaria indicator surveys. Trop Med Infect Dis.

[4] WHO (2018). World malaria report 2018.

[5] Addai-Donkor V. An investigation of factors influencing the uptake of intermittent preventive treatment of malaria in pregnancy programme in the Bekwai Municipality of Ghana. Traditional thesis. Ghana: University for Development Studies; 2015.

[6] Olugbade OT, Ilesanmi OS, Gubio AB, Ajayi I, Nguku PM, Ajumobi O (2019). Socio-demographic and regional disparities in utilization of intermittent preventive treatment for malaria in pregnancy—Nigeria demographic health survey 2013. Pan Afr Med J.

[7] Osei TE (2009). Intermittent preventive treatment of malaria in pregnancy: its effects on maternal morbidity and neonatal birthweight in Offinso District of Ashanti Region, Ghana.

[8] Dellicour S, Tatem AJ, Guerra CA, Snow RW, ter Kuile FO (2010). Quantifying the number of pregnancies at risk of malaria in 2007: a demographic study. PLoS Med.

[9] Amoran OE, Ariba AA, Iyaniwura CA (2012). Determinants of intermittent preventive treatment of malaria during pregnancy (IPTp) utilization in a rural town in Western Nigeria. Reprod Health.

[10] Schantz-Dunn J, Nour NM (2009). Malaria and pregnancy: a global health perspective. Rev Obstet Gynecol.

[11] Hill J, Hoyt J, van Eijk AM, D’Mello-Guyett L, ter Kuile FO, Steketee R (2013). Factors affecting the delivery, access, and use of interventions to prevent malaria in pregnancy in sub-Saharan Africa: a systematic review and meta-analysis. PLoS Med.

[12] Onoka CA, Hanson K, Onwujekwe OE (2012). Low coverage of intermittent preventive treatment for malaria in pregnancy in Nigeria: demand-side influences. Malar J.

[13] Onoka CA, Onwujekwe OE, Hanson K, Uzochukwu BS (2012). Sub-optimal delivery of intermittent preventive treatment for malaria in pregnancy in Nigeria: influence of provider factors. Malar J.

[14] Adeyanju O, Tubeuf S, Ensor T (2017). Socio-economic inequalities in access to maternal and child healthcare in Nigeria: changes over time and decomposition analysis. Health Policy Plan.

[15] Alawode A, Uthman OA, Yahaya I (2012). Socio-economic inequity in accessing malaria control interventions in Nigeria: analysis of changes between 2003 and 2008. Malar J.

[16] United Nations. Transforming our world: the 2030 agenda for sustainable development. United Nations, A/RES/70/1; 2015.

[17] Federal Ministry of Health Nigeria (2018). Second national strategic health development plan 2018–2022.

[18] Uzochukwu B (2017). Primary health care systems: case study from Nigeria.

[19] Nwosu CO, Ataguba JE (2019). Socioeconomic inequalities in maternal health service utilisation: a case of antenatal care in Nigeria using a decomposition approach. BMC Public Health.

[20] Okoli C, Hajizadeh M, Rahman MM, Khanam R (2020). Geographical and socioeconomic inequalities in the utilization of maternal healthcare services in Nigeria: 2003–2017. BMC Health Serv Res.

[21] USA Embassy in Nigeria. Nigeria Malaria Fact Sheet. In: Economic Section. Edited by Nigeria USEi. Nigeria: United States Embassy in Nigeria; 2011. p. 2.

[22] National Population Commission, ICF International (2018). Nigeria Demographic and Health Survey.

[23] Okethwangu D, Opigo J, Atugonza S, Kizza CT, Nabatanzi M, Biribawa C (2019). Factors associated with uptake of optimal doses of intermittent preventive treatment for malaria among pregnant women in Uganda: analysis of data from the Uganda Demographic and Health Survey, 2016. Malar J.

[24] ICF International (2018). Demographic and health surveys standard recode manual for DHS7.

[25] Hill J, Kazembe P (2006). Reaching the Abuja target for intermittent preventive treatment of malaria in pregnancy in African women: a review of progress and operational challenges. Trop Med Int Health.

[26] Iliyasu Z, Gajida AU, Galadanci HS, Abubakar IS, Baba AS, Jibo AM (2012). Adherence to intermittent preventive treatment for malaria in pregnancy in urban Kano, northern Nigeria. Pathog Glob Health.

[27] Atake EH (2021). Socio-economic inequality in maternal health care utilization in sub-Saharan Africa: evidence from Togo. Int J Health Plann Manage.

[28] Hajizadeh M, Hu M, Bombay A, Asada Y (2018). Socioeconomic inequalities in health among Indigenous peoples living off-reserve in Canada: trends and determinants. Health Policy.

[29] O'Donnell O, van Doorslaer E, Wagstaff A, Lindelow M (2008). Analyzing health equity using household survey data: a guide to techniques and their implementation.

[30] Rezaei S, Hajizadeh M, Irandoost SF, Salimi Y (2019). Socioeconomic inequality in dental care utilization in Iran: a decomposition approach. Int J Equity Health.

[31] Hajizadeh M, Sia D, Heymann SJ, Nandi A (2014). Socioeconomic inequalities in HIV/AIDS prevalence in sub-Saharan African countries: evidence from the Demographic Health Surveys. Int J Equity Health.

[32] Wagstaff A (2005). The bounds of the concentration index when the variable of interest is binary, with an application to immunization inequality. Health Econ.

[33] Wagstaff A, Van Doorslaer E, Watanabe N (2003). On decomposingthe causes of health sector inequalities with an application to malnutrition inequalities in Vietnam. J Econometrics.

[34] Chisha Z, Nwosu CO, Ataguba JE (2019). Decomposition of socioeconomic inequalities in cigarette smoking: the case of Namibia. Int J Equity Health.

[35] Pulok MH, van Gool K, Hajizadeh M, Allin S, Hall J (2020). Measuring horizontal inequity in healthcare utilisation: a review of methodological developments and debates. Eur J Health Econ.

[36] StataCorp (2013). Software for statistics and data science. Stata/SE.

[37] Shifti DM, Chojenta C, Holliday EG, Loxton D (2020). Socioeconomic inequality in short birth interval in Ethiopia: a decomposition analysis. BMC Public Health.

[38] Cai J, Coyte PC, Zhao H (2017). Decomposing the causes of socioeconomic-related health inequality among urban and rural populations in China: a new decomposition approach. Int J Equity Health.

[39] Onwujekwe O, Mbachu CO, Ajaero C, Uzochukwu B, Agwu P, Onuh J (2021). Analysis of equity and social inclusiveness of national urban development policies and strategies through the lenses of health and nutrition. Int J Equity Health.

[40] Izugbara CO, Wekesah F (2018). What does quality maternity care mean in a context of medical pluralism? Perspectives of women in Nigeria. Health Policy Plan.

[41] O'Donnell O (2007). Access to health care in developing countries: breaking down demand side barriers. Cad Saude Publica.

