# Supplementary material for: Decomposition of socioeconomic inequalities in the uptake of intermittent preventive treatment of malaria in pregnancy in Nigeria: evidence from Demographic Health Survey
Source: Malar J. 2021 Jul 3;20:300. doi: 10.1186/s12936-021-03834-8 (PMC8254225; doi:10.1186/s12936-021-03834-8)
Supplement: Supplementary file 1 — Additional file 1: Table S1. Description of variables used in the study. [file 12936_2021_3834_MOESM1_ESM.docx]

**Additional file:**

**Table S1: Description of variables used in the study**

| **Variable** | **Variable description** |
| --- | --- |
| Adequate uptake (≥3) of IPTp-SP during pregnancy | 1= if a woman took adequate (≥3) IPTp-SP, 0 otherwise |
| **Demographic variables** |  |
| Woman's age | Woman's age in years |
| *Marital status* |  |
| Married | 1= if a woman is married, 0 otherwise |
| Others | 1= if a woman is single and others, 0 otherwise |
| **Socioeconomic variables** |  |
| *Woman education level* |  |
| No formal education | 1= if a woman has no formal education, 0 otherwise |
| Primary education | 1= if a woman has a primary education, 0 otherwise |
| Secondary education | 1= if a woman has a secondary education, 0 otherwise |
| Higher education | 1= if a woman has higher education, 0 otherwise |
| *Spouse education level* |  |
| No formal education | 1= if a spouse has no formal education, 0 otherwise |
| Primary education | 1= if a spouse has a primary education, 0 otherwise |
| Secondary education | 1= if a spouse has a secondary education, 0 otherwise |
| Higher education | 1= if a spouse has higher education, 0 otherwise |
| *Wealth index* |  |
| Poorest (1) | 1= if a woman is in the poorest quintile, 0 otherwise |
| Poorer (2) | 1= if a woman is in poorer quintile, 0 otherwise |
| Middle (3) | 1= if a woman is in the middle quintile, 0 otherwise |
| Richer (4) | 1= if a woman is in richer quintile, 0 otherwise |
| Richest (5) | 1= if a woman is in the richest quintile, 0 otherwise |
| *Employment status* |  |
| Woman is employed | 1= if a woman is working, 0 otherwise |
| ***Religion*** |  |
| Christian | 1=if a woman is a Christian, 0 otherwise |
| Muslim | 1= if a woman is a Muslim, 0 otherwise |
| Others | 1= if a woman is neither Christian nor Muslim, 0 otherwise |
| **Ecological variable** |  |
| *Place of residence* |  |
| Urban residence | 1= if a woman lives in an urban area (population size larger than 20,000), 0 if she lives in a rural area |
| *Geopolitical zone* |  |
| North-Central | 1= if a woman is from North-Central, 0 otherwise |
| North-East | 1= if a woman is from North-East, 0 otherwise |
| North-West | 1= if a woman is from North-East, 0 otherwise |
| South-East | 1= if a woman is from South-East, 0 otherwise |
| South-South | 1= if a woman is from South-South, 0 otherwise |
| South-West | 1= if a woman is from South-West, 0 otherwise |
| *Distance to a health facility* |  |
| Distance to a clinic is a big problem | 1= if a woman feels that the distance to the nearest clinic is a big problem, 0 if she feels it is not a big problem |
| *Getting medical help for self* |  |
| Permission for self-medical help (big problem) | 1= if a woman feels the need to get permission to go is a big problem, 0 otherwise |
| **Antenatal care variable** |  |
| *Number of ANC visits* |  |
| ≥ 4 times | 1= if a woman made at least four visits, 0 otherwise |

Notes: IPTp-SP **=**Intermittent Preventive Treatment in Pregnancy with Sulfadoxine-pyrimethamine; ANC = Antenatal care.
